# Serum Metabolomics Reveals Metabolic Changes in Freestyle Wrestlers During Different Training Stages

**DOI:** 10.3390/metabo15110737

**Published:** 2025-11-11

**Authors:** Xiaonan Li, Xiangyu Liu, Jianxing Liu, Yinhai Liu, Yumei Han, Wei Zhang

**Affiliations:** 1 School of Physical Education, Shanxi University, Taiyuan 030000, China; lixiaonan@sxu.edu.cn (X.L.);; 2 School of Physical Education, Huainan Normal University, Huainan 232000, China; liuxiangyu@hnnu.edu.cn (X.L.)

**Keywords:** freestyle wrestlers, serum metabolomics, metabolites

## Abstract

**Objectives**: This study aimed to analyze metabolites changes in elite freestyle wrestlers during three specific training phases—pre-training, peak training, and recovery adjustment—through serum metabolomics analyses and biochemical indicator testing, providing preliminary insights for selecting effective functional assessment metrics. **Methods**: Five male wrestlers (20.40 ± 2.07 years) and five female wrestlers (19.60 ± 0.55 years) were enrolled. Morning fasting venous blood samples were collected before training, at peak training intensity, and after training adjustment and recovery. Serum metabolomic analyses using ^1^H nuclear magnetic resonance (^1^H NMR) spectroscopy and assessment of biochemical indicators were performed. **Results**: The metabolomic analysis identified six significantly altered serum biomarkers in male wrestlers and three in females across different training phases. These differential metabolites are primarily implicated in the regulation of energy and amino acid metabolism pathways. Additionally, significant alterations in conventional biochemical indices were observed. **Conclusions**: Metabolomic markers provide a more accurate and comprehensive reflection of metabolic characteristics in freestyle wrestlers, offering a promising complementary approach to traditional biochemical assessments for monitoring physiological states.

## 1. Introduction

Freestyle wrestling, as a high-intensity and intermittent combat sport, is characterized by 2 min rounds of intense competition in which athletes must execute various explosive movements within extremely short bursts while maintaining physical combat [1]. Athletes are required to perform various explosive movements dominated by anaerobic energy supply in a single match, while simultaneously maintaining sustained aerobic loads (≥30% VO_2_ max for ≥5 min) [2,3]. The energetic demands of such activity involve complex transitions between concentric and eccentric muscle actions, reminiscent of the asymmetric energy expenditure observed between positive and negative mechanical work in gradient locomotion [4]. Furthermore, periodic dehydration and dietary restriction programs that are implemented under weight-class systems may increase the risk of muscle catabolism, electrolyte imbalance, and metabolic homeostasis disruption [5], manifesting as metabolic stress characterized by oxidative stress, lactic acidosis, and glycogen depletion. Traditional training monitoring methods can only reflect localized changes in single metabolic pathways, and it is difficult to reveal the overall metabolic response mechanisms involving multiple systems. Therefore, the introduction of systematic analysis methods is urgently needed. Biochemical markers such as blood lactate, creatine kinase, cortisol, and testosterone are commonly used to monitor athletes’ training status. However, these indicators provide isolated snapshots rather than a comprehensive picture of physiological state, making it difficult to reveal the overall metabolic response [6]. Therefore, there is an urgent need to introduce systems-level analytical methods to comprehensively characterize athletes’ metabolic adaptation [7].

Metabolomics is an emerging systems biology research method that offers the technical advantages of high sensitivity and high throughput [8]. With rigorously standardized protocols, it enables the qualitative and quantitative analyses of low-molecular-weight metabolites (<1000 Da) in organisms with good reproducibility, allowing a comprehensive representation of changes in metabolic phenotypes during exercise [9]. In recent years, significant progress has been made in the application of metabolomics technology in the field of athletic training [10]. Belhaj et al. noted that this technology can map the molecular landscape of exercise physiology, enabling precise predictions for training load, fatigue recovery, and personalized nutritional interventions [11]. Heaney et al. found that non-targeted metabolomics analyses can reveal dynamic changes in hundreds of metabolites in serum, urine, or saliva, thereby illuminating real-time states of training intensity, fatigue recovery, and energy metabolism [12]. Existing research has primarily focused on metabolic responses in athletes engaged in endurance sports such as running and swimming [13,14], but there remains a significant gap in metabolomics studies concerning high-intensity intermittent combat sports like wrestling, coupled with a lack of dynamic monitoring data across complete training cycles.

Therefore, this study took elite freestyle wrestlers from Shanxi Province as participants, employing ^1^H NMR metabolomics technology to conduct longitudinal serum analyses over a one-week training period. This comprehensive approach aimed to characterize key metabolic shifts and potential pathway perturbations induced by freestyle wrestling training loads. This not only addresses a gap in systems biology research for combat sports but also provides molecular-level theoretical support for optimizing training cycle design and developing targeted nutritional intervention strategies. It holds practical significance for advancing scientific training in competitive sports.

## 2. Materials and Methods

### 2.1. Participants and Ethical Statement

The study included five female freestyle wrestlers and five male freestyle wrestlers, all of whom held athletic titles of First-Level Athlete or higher, as defined by the Athlete Technical Rank Standards issued by the General Administration of Sport of China. The personal basic information of the participants is shown in Table 1. All participants were in good health, had no history of chronic diseases, and were not undergoing any weight loss or weight gain programs. During the experimental period, all participants resided at the training center and were subject to a standardized schedule and dietary management, refrained from taking any medications or sports supplements affecting metabolism, and received no special interventions. The last training session prior to blood sampling was conducted at least 12 h earlier to minimize acute exercise effects. All participants were informed of the testing procedures and objectives and signed informed consent forms. This experimental protocol was reviewed and approved by the Academic Ethics Committee of Shanxi University, and the approved number was SXULL2024110.

### 2.2. Sample Collection and Storage

**Figure 1:**
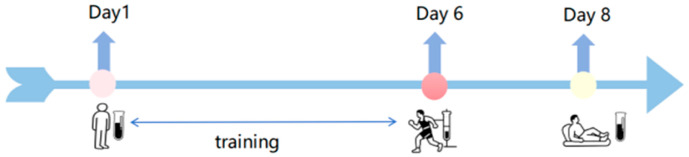
Flow chart of the experimental design.

Participants underwent fasting blood sampling at 7:00 AM via the antecubital vein to collect 7 mL of blood at three distinct time points: pre-training (Day 0), peak training (Day 6), and post-training (Day 8) (Figure 1). One tube (2 mL) was placed in an EDTA anticoagulant tube for whole blood analyses. Another tube (5 mL) was placed in a vacuum blood collection tube. After centrifugation at 3000 r/min for 30 min, the serum was collected and aliquoted into two EP tubes. One tube was used for serum biochemical parameter testing, and the other was stored at −80 °C for subsequent metabolomics analyses.

### 2.3. Measurement of Biochemical Indicators

ABX automated hematology analyzer (Horiba ABX, Montpellier, France) was used to measure white blood cell count (WBC), red blood cell count (RBC), hemoglobin (HB), hematocrit (HCT), and mean corpuscular volume (MCV). Kits (Biosino Bio-Technology and Science Incorporation, Beijing, China) were employed to detect creatine kinase (CK), blood urea nitrogen (BUN), and testosterone (T). Testing was performed according to the methods specified in the instruction manual.

### 2.4. Metabolomics Analyses Methods

#### 2.4.1. H-NMR Sample Preparation

After thawing serum samples at 4 °C, 500 μL of serum was aliquoted into a microcentrifuge tube. Then, 350 μL of D_2_O was added, followed by centrifugation at 4 °C and 18,000× *g* for 20 min. A total of 600 μL of the supernatant was transferred to an NMR tube for spectral acquisition using a Bruker 600 MHz spectrometer (Bruker BioSpin GmbH, Rheinstetten, Germany) at 25 °C.

#### 2.4.2. Spectral Data Processing and Analyses

The serum NMR spectra were processed using MestReNova 6.1.0 software. After Fourier transformation, chemical shifts were calibrated to the trimethylsilylpropanoic acid (TSP) peak (δ 0.00). A standardized iterative protocol was applied for manual baseline and phase correction to ensure flat baselines and symmetrical peaks, minimizing bias. The spectra were segmented into bins of 0.04 ppm across the range of 0.5–10.0 ppm. To avoid interference from the residual water signal, the region between 4.5 and 4.7 ppm was excluded. To validate multivariate models, R^2^Y, Q^2^, and permutation tests (200 permutations) were performed, confirming model robustness and minimal overfitting. Finally, the areas of all integral values are normalized and exported to Excel, yielding a matrix of each segment and its corresponding integral area value. Metabolite identification followed the Metabolomics Standards Initiative (MSI) guidelines.

### 2.5. Statistical Analyses

Data are expressed as mean ± SD. Statistical analyses were performed using SPSS 18.0 (LLC, San Diego, CA, USA). Graphs were generated with GraphPad Prism 7.0 (IBM, Chicago, IL, USA). Multivariate statistical analyses were conducted with SIMCA-P 14.1 (Umetrics, Malmö, Sweden). Principal component analysis (PCA) was first applied to observe intrinsic clustering and outliers. Subsequently, partial least squares-discriminant analyses (PLS-DA) and orthogonal partial least squares-discriminant analyses (OPLS-DA) were employed to enhance group separation and identify differentially expressed metabolites. Metabolites with variable importance in projection values (VIP) greater than 1 and with *p*-values less than 0.05 were considered statistically significant.

## 3. Results

### 3.1. Results of Biochemical Indicator Tests

Table 2 presents the biochemical indicator test results for athletes of different sexes across various training phases. Male wrestlers showed significantly higher concentrations of CK and BU during peak training periods compared to pre-training, with the concentrations of CK significantly decreasing post-training. Compared with pre-training, female wrestlers showed significantly increased serum concentrations of CK (*p* < 0.05) and significantly decreased concentrations of T (*p* < 0.05) during the peak training period. After adequate rest, their CK concentrations significantly decreased following post-training.

### 3.2. Results of Serum Metabolomics Detection

#### Assignment and Analyses of ^1^H-NMR Spectra

**Figure 2:**
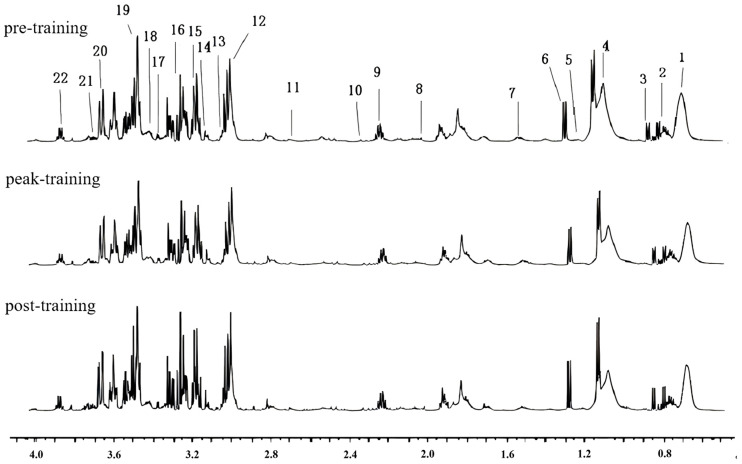
^1^H-NMR spectra of serum metabolites in athletes at different training stages.

The main compounds in the serum sample spectra of athletes were assigned by analyzing data such as chemical shifts, coupling constants and peak patterns, combined with public databases including the Human Metabolome Database (HMDB, http://www.hmdb.ca/) and the Biological Magnetic Resonance Data Bank (BMRB, https://bmrb.io), as well as relevant literature reports. A total of 22 metabolites were identified (Figure 2, Table 3), mainly including amino acids, carbohydrates, and nitrogen-containing compounds.

### 3.3. Formatting of Mathematical Components

#### Results of Multivariate Statistical Analyses

**Figure 3:**
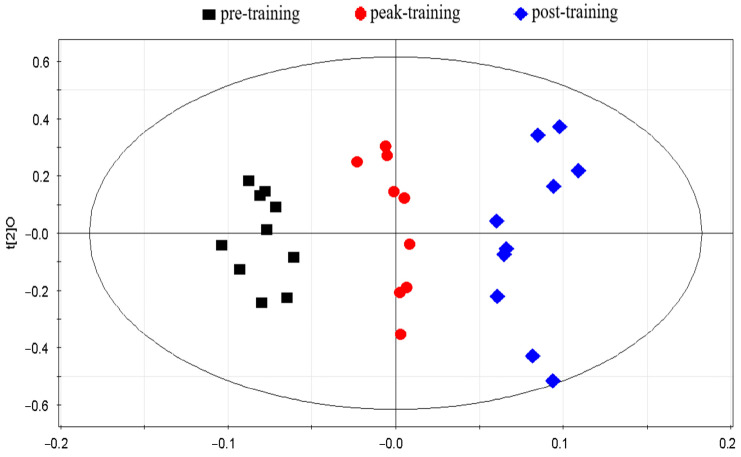
Score plot of PLS-DA analyses.

Further model validation analyses using PLS-DA on serum samples from 10 freestyle wrestlers at different training stages resulted in the score plot shown in Figure 3. It is evident that the three groups of serum samples, namely pre-training, peak training, and post-training, are clearly separated, while samples within the same group achieved clustering.

To further identify metabolite differences between male wrestlers and female wrestlers across distinct training phases, athletes were categorized by sex. Metabolites were comparatively analyzed for male and female wrestlers before training, during peak training, and after training, resulting in OPLS-DA score plots and loading plots. For compounds with VIP ≥ 1 in the loading plot, one-way ANOVA was performed on their corresponding metabolite peak areas using SPSS software. This ultimately identified the significantly different metabolites (*p* < 0.05).

**Figure 4:**
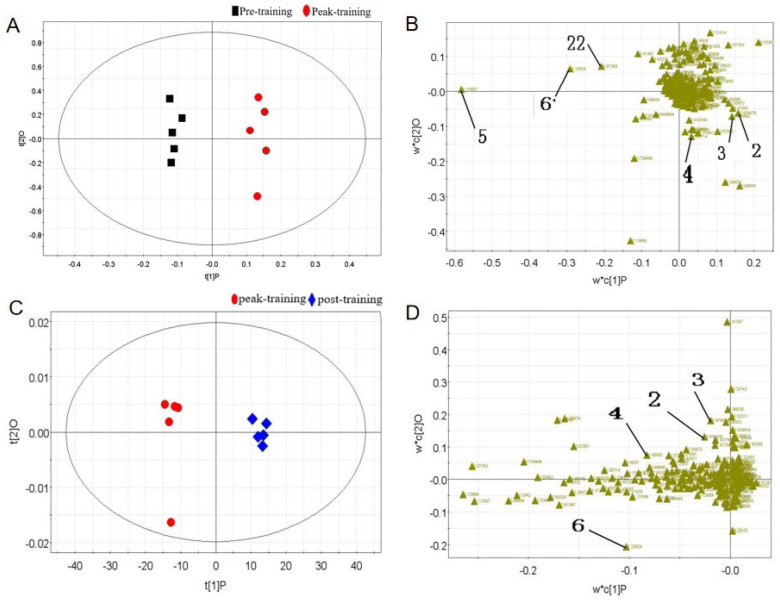
Multivariate data analyses of male wrestlers. (**A**) OPLS-DA score plot from pre-training and peak training; (**B**) load plot from pre-training and peak training; (**C**) OPLS-DA score plot from peak training and post-training; (**D**) load plot from peak training and post-training. 2: Leucine, 3: Isoleucine, 4: Valine, 5: β-hydroxybutyric acid, 6: Lactic acid, 22: Hippurate.

Compared to pre-training, a total of six differential metabolites were identified in the serum of male wrestlers in the peak training (Figure 4) (Table 4). Among these, the concentrations of leucine, isoleucine, valine, and lactate showed significant increases, while the concentrations of β-hydroxybutyrate and hippurate showed significant decreases. After adequate rest, the concentrations of leucine, isoleucine, valine, and lactate in male wrestlers decreased significantly compared to the peak training.

**Figure 5:**
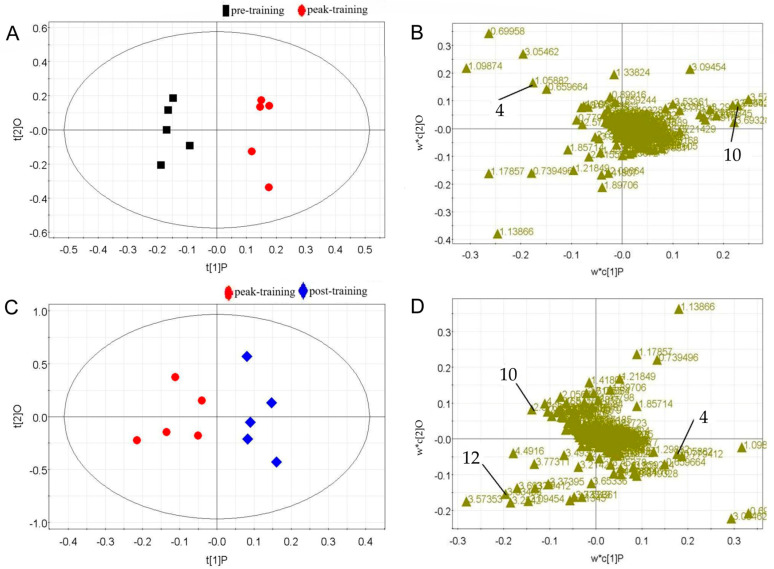
Multivariate data analyses of female wrestlers. (**A**) OPLS-DA score plot from pre-training and peak training; (**B**) load plot from pre-training and peak training; (**C**) OPLS-DA score plot from peak training and post-training; (**D**) load plot from peak training and post-training. 4: Valine, 10: Pyruvate, 12: Creatine.

Compared to pre-training, a total of three differential metabolites were identified in the serum of female wrestlers during peak training (Figure 5) (Table 5). Among these, pyruvate concentrations showed a significant increase, while valine concentrations exhibited a significant decrease (*p* < 0.05). After adequate rest, the concentrations of pyruvate decreased significantly, while those of creatine and valine increased significantly (*p* < 0.05) compared to the peak training.

**Figure 6:**
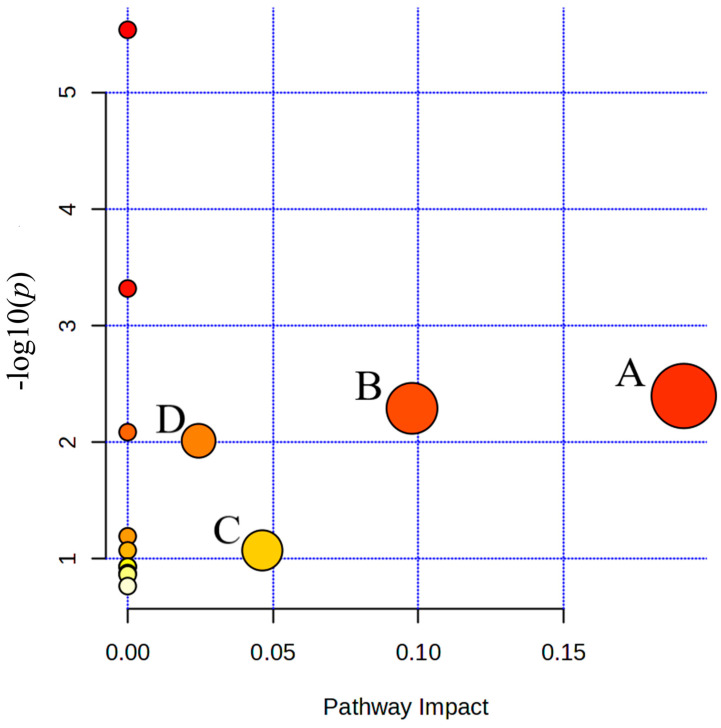
Summary diagram of pathway analyses with Met-PA. (A) Pyruvate metabolism; (B) Glycolysis or Gluconeogenesis; (C) Citrate cycle (TCA cycle); (D) Arginine and proline metabolism.

To further identify the metabolic pathways affected by the potential serum biomarkers in freestyle wrestlers, all screened potential biomarkers were imported into the online analysis platform MetaboAnalyst 5.0 (http://www.metaboanalyst.ca/), and a pathway impact plot was generated (Figure 6). The key metabolic pathways involved were derived from this plot. As shown in Table 6, freestyle wrestling training primarily affected four metabolic pathways: pyruvate metabolism, glycolysis or gluconeogenesis, citrate cycle (TCA cycle), and arginine and proline metabolism.

## 4. Discussion

This study analyzed serum samples collected from freestyle wrestlers during different training phases of the intensive training cycle. By integrating conventional biochemical indicators with metabolomics technology, it explored the metabolic response characteristics and recovery status of athletes’ bodies to training loads.

### 4.1. Training Load and Recovery Status Reflected by Conventional Biochemical Indicators

Conventional biochemical indicators serve as a critical window for evaluating exercise load and bodily adaptability [15]. This study found that both male wrestlers and female wrestlers showed a significant increase in the concentration of CK at the peak training compared to pre-training. CK serves as a sensitive indicator reflecting the extent of muscle damage and the intensity of exercise load [16]. The significant elevation of CK in athletes indicates that the training during this stage exerted substantial mechanical stimulation on their skeletal muscles, achieving the expected effect of targeted training intensity [17]. Notably, after an appropriate rest period, CK concentrations decreased significantly, suggesting that the athletes possessed a favorable short-term recovery capacity.

The changes in the concentration of BU mainly reflect the state of protein catabolism caused by exercise consumption and the body’s potential for recovery [18]. Normally, the concentration of BU changes little during short-duration exercise (<30 min), with significant increases primarily observed after prolonged or high-intensity exercise [19]. It was found that an increase in the concentration of BU exceeding 3 mmol/L before and after training indicates excessive training volume leading to fatigue accumulation. An increase of approximately 2 mmol/L suggests a moderate training volume, while an increase of about 1 mmol/L indicates relatively insufficient training volume [20]. In this study, the concentration of BU in male wrestlers at peak training increased by only 1.15 mmol/L compared to pre-training, indicating that the training volume was relatively low. It suggests that the training load in the current phase is insufficient to effectively stimulate the bodies of male wrestlers, failing to fully activate deep physiological adaptation mechanisms.

Furthermore, the T concentration of female wrestlers showed a significant decreasing trend at the training peak. Testosterone is not only a key hormone reflecting anabolic status and bodily recovery capacity, but also plays a crucial role in enhancing the excitability of the central nervous system [21,22]. It can activate the protein synthesis enzyme system, and stimulate muscle cell receptors to promote muscle repair and growth [23]. Some studies showed that the serum concentration of T decreased by more than 25% after exercise and failed to recover after training, suggesting that athletes were not adapting to the training load and might be experiencing excessive fatigue, which required adjustments to the training schedule [24]. This study found that female wrestlers showed a significant decrease in the concentration of T during the peak training period compared to pre-training, exceeding the warning threshold. This indicates that the training load during this phase stimulated the athletes’ bodies to a deeper degree, potentially approaching the upper limit of their capacity. Notably, after adequate rest, there was a significant rebound in the concentration of T among the female wrestlers. This suggests that although the load was substantial, it remained within the athletes’ controllable range, and their bodies possessed a certain potential for recovery.

### 4.2. Sex-Specific Metabolic Response Patterns Revealed by Metabolomics

Metabolomics analyses further revealed the complex molecular-level responses of athletes to training stress and demonstrated significant sex differences.

#### 4.2.1. Metabolic Response Characteristics of Male Wrestlers

A total of 6 serum metabolic biomarkers were identified to significantly change across the different training phases in male wrestlers: leucine, isoleucine, valine, β-hydroxybutyrate, lactate, and hippuric acid. Compared to pre-exercise, the concentrations of leucine, isoleucine, and valine (BCAAs) and lactate significantly increased at peak training, while the concentrations of β-hydroxybutyrate and hippuric acid significantly decreased. Compared to the peak training period, the concentrations of leucine, isoleucine, valine, and lactate decreased significantly post-exercise; there was no significant change in the concentrations of β-hydroxybutyrate and hippuric acid.

BCAAs, as essential amino acids, cannot be synthesized by the human body and must be obtained through dietary intake. During prolonged or high-intensity exercise, they undergo extensive catabolic metabolism in muscle tissue to provide energy [25]. Leucine metabolism can generate ketone bodies, isoleucine generates ketone bodies and acyl-CoA, while valine can ultimately generate glucose [26]. This study found that male wrestlers exhibited elevated BCAA concentrations during peak-training, contrasting with the declining BCAA trend observed in endurance-based prolonged exercise [27]. This discrepancy may be attributed to the intermittent, explosive nature of wrestling, which drives protein breakdown rates beyond oxidative utilization [28]. This finding is also consistent with the transient elevation of BCAA concentrations during anaerobic sprinting, suggesting sport-specific metabolic characteristics across different athletic disciplines [29]. Notably, the occurrence of exercise-induced fatigue is closely related to BCAA metabolism [30]. Prolonged high-load exercise can alter the plasma amino acid profile, increasing the ratio of AAAs to BCAAs [31]. This imbalance is believed to accelerate the transport of amino acids (particularly tryptophan) into the brain, promote serotonin synthesis, enhance central inhibition, and thereby induce central fatigue [32]. Therefore, the elevated serum BCAA concentrations observed at peak training in this study reflect their mobilization as energy substrates.

Lactic acid is the end product of glycolysis [33]; its significant increase at the peak-training directly confirms that anaerobic glycolysis is one of the primary energy supply pathways for male wrestlers during wrestling training. Hippurate is synthesized in the liver from benzoate, which is largely derived from gut microbiota metabolism [34,35]. In this study, hippurate significantly decreased at the training peak and did not rebound significantly during recovery. This decrease at the peak-training may be related to exercise-induced alterations in gut microbial composition or activity [36]. The lack of significant rebound during post-training may indicate the long-term effects of training load on this gut–liver metabolic axis [37].

#### 4.2.2. Metabolic Response Characteristics of Female Wrestlers

A total of three significantly different metabolic markers were identified in female wrestlers across different training phases: valine, pyruvate, and creatine, with fewer markers identified compared to male wrestlers. Compared to pre-training, there was a significant decrease in the concentration of valine and a significant increase in the concentration of pyruvate at the peak training, while the concentration of creatine showed no significant change. Compared to peak training, the concentration of valine and creatine significantly increased post-training, while the concentration of pyruvate significantly decreased.

Valine is an important glucogenic amino acid, and its metabolism ultimately generates glucose [38]. Unlike the increasing trend observed in male wrestlers, female wrestlers showed a significant decrease in the concentration of valine in serum during peak training. This reveals sex differences in energy metabolism. The decline in the valine concentrations of female wrestlers indicates its rapid uptake and breakdown for energy to meet the demands of high-intensity training. This finding correlates with the significantly reduced the concentration of T observed in female wrestlers in this study, suggesting the body is in a more pronounced catabolic state during this phase [39]. Research indicates that women often rely more heavily on fat oxidation during exercise [40]. However, during high-intensity interval training (HIIT) with substantial glycogen depletion, they may instead enhance amino acid gluconeogenesis, leading to increased consumption of valine [41]. This suggests the need to focus on the intake and supplementation of BCAAs for female wrestlers to support training and delay fatigue. Pyruvic acid is a key intermediate product of glucose metabolism [42]. The significantly increased concentration of pyruvate at peak training reflects the overall activation of energy metabolism pathways to meet training demands [43].

Unlike male wrestlers, this study did not observe a significant increase in the concentration of lactate at peak training in female wrestlers. Since lactic acid is converted from pyruvic acid under hypoxic conditions [44], this finding indicates that during the training phase, female wrestlers show relatively lower activity in the glycolytic energy pathway compared to male wrestlers, or in other words, a smaller proportion of pyruvate flows into the lactate pathway. Combined with the significant decrease in the concentration of valine, this further supports that catabolism plays a substantial role in energy supply for female wrestlers during this phase. There was no significant change in the concentration of creatine at peak training intensity, but it increased significantly after training. Creatine participates in the phosphocreatine system and energy buffering [45]. Female wrestlers showed significantly increased serum creatine concentrations post-training, potentially due to active uptake and resynthesis of creatine by muscle cells to prepare for subsequent energy demands. Alternatively, this may reflect an overall improvement in the anabolic environment, promoting endogenous creatine synthesis [46].

#### 4.2.3. Pathway Perturbations and Training Adaptation

The serum metabolomic profiles effectively capture the comprehensive physiological demands of freestyle wrestling training across different phases. The significantly elevated concentrations of lactate and pyruvate, particularly in male wrestlers, directly confirm the activation of anaerobic glycolysis as a primary energy pathway during high-intensity efforts [47,48].

The alterations in branched-chain amino acids (BCAAs) reveal distinct sex-specific metabolic responses. The increased BCAA concentrations in male wrestlers suggest a sport-specific pattern where the rate of protein catabolism and BCAA mobilization exceeds their oxidative utilization [49]. In contrast, the decreased valine concentration in female wrestlers indicates enhanced utilization of glucogenic amino acids for energy production, highlighting fundamental differences in substrate utilization between sexes [50].

The observed changes in other metabolites provide additional insights into metabolic adaptation. The decreased β-hydroxybutyrate concentration during peak training suggests a redirection of acetyl-CoA from ketogenesis toward the TCA cycle to meet energy demands [51]. Meanwhile, the significantly increased creatine concentration in female wrestlers during recovery indicates active replenishment of the phosphocreatine system, reflecting an anabolic response following training stress [52].

### 4.3. Limitations of the Study

This study has several limitations that should be considered when interpreting the results. First, the relatively small sample size and the exclusive inclusion of elite-level wrestlers may limit the generalizability of the findings to athletes of different competitive levels or training backgrounds. However, a post hoc power analysis was conducted based on the observed large effect sizes in biochemical markers (e.g., Creatine Kinase, Cohen’s d > 3.0) and the clear separation in multivariate models. The analysis indicated that for detecting changes of such magnitude, the sample size provided a statistical power (1-β) exceeding 0.99, which is well above the conventional threshold of 0.80. This suggests that the study was sufficiently powered to identify the primary, large-effect metabolic and physiological responses reported. Nevertheless, the small sample size remains a constraint for detecting subtle effects, ensuring robust generalizability, and for conducting more complex subgroup analyses.

Second, although ^1^H-NMR metabolomics provides high reproducibility and is suitable for untargeted profiling, its comparatively lower sensitivity than mass spectrometry-based approaches may have led to the under-detection of certain low-abundance metabolites that could be relevant to exercise-induced metabolic adaptations.

Third, despite efforts to standardize the participants’ diet and living conditions, potential confounding factors—such as individual nutritional intake, hydration status, and, for female athletes, menstrual cycle phase—were not strictly monitored or controlled. These factors may have introduced additional variability into the metabolic data.

Finally, the metabolic profiling was performed over a single training microcycle. Although this approach offers valuable insights into short-term dynamics, it does not reflect long-term adaptive responses or potential metabolic periodization across a full training macrocycle. Future longitudinal studies involving larger cohorts, integrated multi-omics methodologies, and detailed workload monitoring are warranted to validate and extend these preliminary findings.

## 5. Conclusions

Serum differential metabolites in freestyle wrestlers across different training phases were primarily enriched in four metabolic pathways: pyruvate metabolism, glycolysis or gluconeogenesis, citrate cycle (TCA cycle), and arginine and proline metabolism. These pathways predominantly regulate energy metabolism and amino acid metabolism, suggesting metabolic adaptations under exercise load. This indicates that relevant metabolic biomarkers may serve as new targets for assessing athletes’ metabolic status and the extent of recovery.

This study combines traditional biochemical monitoring with metabolomics analyses to provide a multiple-aspect perspective on the physiological functions and metabolic characteristics of freestyle wrestlers. The information of small-molecule metabolites detected by ^1^H NMR metabolomics reflects the training load and physiological status of athletes of different sexes, providing a basis for training monitoring and scientific training. Future studies may explore larger sample sizes and multimodal monitoring to further illuminate the relationship between metabolites, exercise fatigue, and training load, thereby enabling a more comprehensive assessment of wrestling’s metabolic characteristics.

## Figures and Tables

**Table 1:** Basic information of participants.

Sex	Age (Years)	Height (cm)	Weight (kg)
Men	20.40 ± 2.07	180.20 ± 3.83	88.60 ± 15.66
Women	19.60 ± 0.55	164.20 ± 4.02	64.40 ± 4.51

**Table 2:** Blood biochemical parameters of athletes by sex.

Parameter	Male Wrestlers			Female Wrestlers		
	Pre-Training	Peak Training	Post-Training	Pre-Training	Peak Training	Post-Training
WBC (×109 g/L)	5.40 ± 0.88	4.70 ± 0.42	5.04 ± 0.72	5.92 ± 1.76	5.14 ± 1.19	4.96 ± 1.07
RBC (×1012 g/L)	4.76 ± 0.29	4.48 ± 0.40	4.83 ± 0.37	4.09 ± 0.16	3.95 ± 0.19	4.14 ± 0.32
Hb (g/L)	155.80 ± 8.93	151.20 ± 9.15	160.20 ± 8.44	133.40 ± 6.35	126.80 ± 8.58	134.00 ± 11.77
Hct (%)	45.46 ± 1.40	43.02 ± 2.53	45.80 ± 2.08	38.46 ± 1.70	37.02 ± 1.77	38.96 ± 3.06
MCV (Fl)	95.60 ± 3.08	95.20 ± 3.30	95.06 ± 3.59	94.02 ± 3.62	93.68 ± 2.95	94.08 ± 3.06
CK (U/L)	183.60 ± 48.44	451.80 ± 95.70 *	228.00 ± 48.84 #	80.80 ± 14.51	230.00 ± 29.80 *	98.40 ± 32.19 #
BU (mmol/L)	5.10 ± 0.43	6.25 ± 0.30 *	5.58 ± 0.83	4.87 ± 1.89	6.78 ± 1.66	5.27 ± 1.52
T (ng/dL)	666.60 ± 101.9	554.40 ± 135.07	580.40 ± 146.26	50.35 ± 12.94	34.17 ± 12.27 *	36.26 ± 15.35

* *p* < 0.05 vs. Pre-training; # *p* < 0.05 vs. Peak Training.

**Table 3:** Assignment results of serum metabolites in athletes via ^1^H-NMR spectra.

No.	Compound Name	Chemical Shift (δ)	Peak Shape
1	Lipids	0.89	m
2	Leucine	0.95	d
3	Isoleucine	0.99	d
4	Valine	1.04	d
5	β-Hydroxybutyrate	1.15	dd
6	Lactic Acid	1.32	d
7	Alanine	1.48	d
8	N-Acetylglycoproteins	2.04	s
9	Acetoacetic Acid	2.27	s
10	Pyruvic Acid	2.37	s
11	Citric Acid	2.65	d
12	Creatine	3.03	s
13	Creatinine	3.04	t
14	Choline	3.20	s
15	Trimethylamine N-Oxide	3.23	m
16	Taurine	3.26	t
17	Proline	3.41	d
18	α-Glucose	3.47	d
19	Glycerol	3.53	s
20	β-Glucose	3.71	d
21	Glutamic Acid	3.72	t
22	Hippurate	3.98	d

s: single peak, d: double peak, dd: two double peaks, t: triple peak, m: multiple peaks.

**Table 4:** Results of differentially metabolites in serum of male wrestlers.

No.	Metabolites	Chemical Shift (δ)	Mean ± SD	Peak-Training vs. Pre-Training	Post-Training vs. Peak-Training
1	Leucine	0.95 (d)	0.0009 ± 0.00054	↑	↓
2	Isoleucine	0.99 (d)	0.0009 ± 0.00050	↑	↓
3	Valine	1.05 (d)	0.0030 ± 0.00052	↑	↓
4	β-hydroxybutyric acid	1.17 (dd)	0.0698 ± 0.00989	↓	—
5	Lactic acid	1.32 (d)	0.0116 ± 0.0278	↑	↓
6	Hippurate	3.98 (d)	0.0060 ± 0.00112	↓	—

d: double peaks, dd: two double peaks; ↑: increase, ↓: decrease, —: no significant change.

**Table 5:** Results of differentially metabolites in serum of female wrestlers.

No.	Metabolites	Chemical Shift (δ)	Mean ± SD	Peak- Training vs. Pre-Training	Post-Training vs. Peak Training
1	Valine	1.05 (d)	0.0082 ± 0.00123	↓	↑
2	Pyruvate	2.37 (s)	0.0016 ± 0.00040	↑	↓
3	Creatine	3.03 (s)	0.0389 ± 0.00326	—	↑

d: double peaks, s: single-peak; ↑: increase, ↓: decrease, —: no significant change.

**Table 6:** Pathway enrichment analyses results from MetaboAnalyst 5.0.

Metabolic Pathway	Total	Hits	*p*	−log(*p*)	Holm *p*	FDR	Impact
Pyruvate metabolism	23	2	0.004014	2.3964	0.31309	0.10248	0.19137
Glycolysis or Gluconeogenesis	26	2	0.0051238	2.2904	0.39453	0.10248	0.09785
Citrate cycle (TCA cycle)	20	1	0.084848	1.0714	1.0	0.7542	0.04634
Arginine and proline metabolism	36	1	0.0097248	2.0121	0.72936	0.12966	0.02442

## Data Availability

The original contributions presented in this study are included in the article. Further inquiries can be directed to the corresponding authors.
